# A Precision Medicine Model for Targeted Antibiotic Therapy in Urinary Tract Infections: A Valuable Tool to Reduce Hospitalization Stay and the Time to Switch to Oral Treatment

**DOI:** 10.3390/antibiotics14020211

**Published:** 2025-02-19

**Authors:** Tommaso Cai, Anna Brugnolli, Massimiliano Lanzafame, Fabiana Dellai, Carlo Tascini, Claudio Scarparo, Vito Racanelli, Orietta Massidda, Gernot Bonkat, Luca Gallelli, Truls E. Bjerklund Johansen

**Affiliations:** 1 Department of Urology, Santa Chiara Regional Hospital, 38123 Trento, Italy; 2 Institute of Clinical Medicine, University of Oslo, 0025 Oslo, Norway; t.e.b.johansen@medisin.uio.no; 3 Centre of Higher Education for Health Sciences, 38123 Trento, Italy; anna.brugnolli@apss.tn.it; 4 Centre for Medical Sciences (CISMed), University of Trento, 38123 Trento, Italy; massimiliano.lanzafame@apss.tn.it (M.L.); vito.racanelli@apss.tn.it (V.R.); orietta.massidda@untn.it (O.M.); 5 Department of Infectious Diseases, Santa Chiara Regional Hospital, 38123 Trento, Italy; 6 Infectious Diseases Clinic, Department of Medicine (DAME), University of Udine, 33100 Udine, Italy; dellai.fabiana@spes.uniud.it (F.D.); carlo.tascini@uniud.it (C.T.); 7 Department of Microbiology, Santa Chiara Regional Hospital, 38123 Trento, Italy; claudio.scarparo@apss.tn.it; 8 Internal Medicine Division, Santa Chiara Hospital, Provincial Health Care Agency (APSS), 38123 Trento, Italy; 9 Department of Cellular, Computational and Integrative Biology (CIBIO), University of Trento, 38123 Trento, Italy; 10 alta uro AG, Merian Iselin Klinik, Center of Biomechanics & Calorimetry, University of Basel, 4001 Basel, Switzerland; bonkat@alta-uro.com; 11 Department of Health Science, School of Medicine, University of Catanzaro, 88100 Catanzaro, Italy; gallelli@unicz.it; 12 Department of Urology, Oslo University Hospital, 0025 Oslo, Norway; 13 Institute of Clinical Medicine, University of Aarhus, 8210 Aarhus, Denmark

**Keywords:** precision medicine, urinary tract infection, antibiotic stewardship, risk factors, antibiotic resistance

## Abstract

**Background/Objectives**: The management of urinary tract infections (UTIs) has become an increasingly challenging medical intervention. This study explores whether adoption of a precision medicine model could improve the management of acute uncomplicated pyelonephritis (uAPN) or complicated UTIs (cUTIs) compared with the standard of care approach, in hospitalized patients. **Methods**: From January 2022 to March 2024, all patients affected by uAPN or cUTIs and attending our urological institution were randomized to receive the following: antibiotic treatment according to guidelines and recommendations (standard of care group) or antibiotic treatment according to the precision medical model (intervention group). The main outcome measures were the rates of clinical success and the length of hospitalization. The time until switching to oral treatment was regarded as a secondary outcome measure. **Results**: Eighty-three patients were enrolled in the standard of care group, while seventy-nine patients were enrolled in the intervention group. While the overall clinical success rate was similar in the two groups (75 vs. 72; *p* = 0.97), a statistically significant difference was observed between the two groups in terms of length of hospitalization (8 days vs. 5 days; *p* = 0.03) and time to switch to oral treatment (96 h vs. 72 h; *p* = 0.04). A statistically significant difference was found between the two groups regarding the need to change antimicrobial therapy during hospitalization [12 out of 80 vs. 6 out of 77; *p* = 0.04]. **Conclusions**: Adoption of the precision medicine model appears as a valuable means to improve the management of patients with uAPN and cUTIs. By reducing the period of hospitalization and the time to switch to oral treatment, the precision medicine model also improves antimicrobial stewardship in the management of UTIs.

## 1. Introduction

The management of urinary tract infections (UTI) represents one of the most frequent medical interventions both in hospital and outpatient settings, even if clinical results are often unsatisfactory with relapses, recurrences, and progression to more severe types of infections [1]. Despite increased involvement of infectious disease specialists and clinical microbiologists, we have not been able to reduce the rate of recurrences, and progression to more severe infections [2,3]. Today, UTI management has become increasingly challenging and the EAU guidelines on UTIs is now the second most visited site on Uroweb, next to prostate cancer. Recently, we have witnessed the adoption of precision medicine in various fields of medicine, fueled by scientific as well as political arguments [4]. We therefore observed several arguments for adopting the principles of precision medicine also in the management of UTIs, such as in the relationship between gene expression and the risk of recurrent uncomplicated UTIs or severe infections [5]. Moreover, it has been clearly demonstrated that a patient`s lifestyle is associated with risk of UTI, especially in the presence of other risk factors [6]. The epidemiology of antimicrobial resistance (AMR) differs significantly among countries and regions. This means that we should not treat a certain type of UTI with the same antibiotics in areas with different prevalences of pathogens and antimicrobial resistance. In the era of antimicrobial stewardship, we need a new and more comprehensive and differentiated approach to the management of UTIs, and we believe that adoption of the precision medicine model could help us achieve this goal. In principle, our precision model means that we pay more attention to a patient’s personal history and risk factors in our treatment decisions as opposed to automatically following the order of antibiotic recommendations in international guidelines. Here, we aim to explore whether antibiotic treatment according to a precision medicine approach could help us improve the management of hospitalized patients with acute uncomplicated pyelonephritis (uAPN) or complicated UTIs (cUTIs) as compared with the standard of care according to guidelines.

## 2. Results

From an initial cohort of 181 patients admitted to our center between January 2022 and March 2024 with suspected uAPN or cUTI, 162 patients were eligible for the study and treated according to the group assignment: 83 patients were treated according to the standard of care while 79 patients were treated according to the precision medicine model. Five patients were excluded from the final analysis due to missing data. Finally, 80 patients in the standard of care group and 77 patients in the intervention group concluded the study and their data were analyzed (Table 1). All patients were correctly treated in the two groups according to protocol and guidelines.

### 2.1. Empirical Antibiotic Treatment

In the standard of care group, the most commonly prescribed antibiotics were piperacillin/tazobactam, followed by cephalosporins and fluoroquinolones. This prescription pattern might be due to either a limitation in the guidelines, which recommend wide spectrum antibiotics without taking the patient’s risk factors into account, or a fear of ESBL strains. In the intervention group, the most commonly prescribed antibiotics were amoxicillin-clavulanic acid, followed by cephalosporins and aminoglycoside. Table 1 shows all antibiotics prescribed at hospital admission in each study group.

### 2.2. Clinical and Microbiological Success Rates

In the standard of care group, we observed overall clinical success in 75 patients out of 80 (93.7%). In the intervention group, we observed overall clinical success in 72/77 patients (93.5%). No statistically significant difference was found between the two groups (*p* = 0.97). Regarding the microbiological results, 76 out of 80 patients (95.0%) in the control group and 74 out of 77 patients (96.1%) in the intervention group had a microbiological success with negative urine—and blood culture. No statistically significant difference was found between the two groups in terms of microbiological success (*p* = 0.99). Table 2 shows the clinical and microbiological results at the follow-up evaluation for each study group. No statistically significant difference has been reported between the two groups in terms of *Clostridioides difficile* isolation (1.21% vs. 1.25% *p* = 1.0). No adverse events were registered in any of the two groups.

### 2.3. Need for Re-Evaluation and Change of Antibiotic Treatment During the Hospital Stay

During the hospital stay, 12 out of 80 patients (15%) in the standard of care group required an antibiotic treatment change, especially among patients treated with fluoroquinolones. On the other hand, 6 out of 77 patients (7.8%) in the intervention group required an antibiotic treatment re-evaluation and change due to a lack of clinical response or inflammatory marker response. A statistically significant difference was found between the two groups in terms of antimicrobial therapy change during hospitalization (*p* = 0.04).

### 2.4. Switch to Oral Treatment

A switch to oral treatment was registered in all patients. In the standard of care group, the median time to a switch of treatment was 96 ± 8 h (95% CI 94.2–97.7), as compared to a median of 72 ± 4 h in the intervention group (95% CI 71.1–72.8). A statistically significant difference was found between the two groups in the time before switching to oral treatment (*p* < 0.001). The patients who had the longest time to treatment switch were those who underwent piperacillin/tazobactam therapy (92 ± 3) irrespective of study group affiliation.

### 2.5. Length of Hospitalization

The median length of hospitalization was 8 ± 3 (95% CI 7.3–8.6) days in the standard of care group versus 5 ± 2 (95% CI 4.5–5.4) days in the intervention group. This difference is also highly statistically significant (*p* < 0.001). The length of hospitalization was shorter for the intervention group due to the shorter amount of time before switching to oral treatment.

## 3. Discussion

### 3.1. Major Findings

In this study, we demonstrated that adoption of a precision medicine model improves the management of APN or cUTIs in terms of length of hospitalization and time before switching to oral treatment. Moreover, the use of this model may reduce the need for re-evaluation and change of antibiotic treatment during hospital stay. Our precision medicine model, which is based on clinical characteristics and anamnestic data, allowed us to reduce prescriptions of piperacillin/tazobactam, which should be reserved for severe infections. In fact, the most commonly used antibiotic in the intervention group was amoxicillin-clavulanic acid, instead of piperacillin/tazobactam. Moreover, even if the clinical efficacy in the intervention group was not superior to the standard of care group, our model allowed us to reduce the need for antimicrobial therapy changes during hospitalization and reduced the time before switching to oral treatment. Thus, the model was able to reduce the patients’ exposure to broad-spectrum antibiotics and remains important for compliance with antimicrobial stewardship principles in urology. The similar clinical and microbiological success rates between groups despite shorter hospitalization times and fewer antibiotic switches in the intervention group warrant interesting considerations beyond the scope of the present paper.

### 3.2. Results in Comparison with Other Studies

Watkins RR, recently highlighted precision antimicrobials as an alternative to broad spectrum antibiotics [7]. He advocated for new targeted therapeutic strategies, including narrow-spectrum agents, engineered probiotics, nanotechnology, phage therapy, and CRISPR-Cas9 technology [8]. A deeper comprehension of the benefits, disadvantages, and modes of action of these new treatments can help urologists enhance current antibacterial strategies and explore new treatment opportunities [8]. Recently, Italian health authorities recommended the use of The WHO AWaRe (Access, Watch, Reserve) book by the WHO to guide the correct choice of antibiotics in everyday clinical practice [9]. According to this manual, piperacillin/tazobactam has been inserted in the “Watch” class in comparison with amoxicillin-clavulanic acid, which has been included in the “Access” class. In this sense, the precision medicine model facilitates a choice of antibiotic according to the recommendation of WHO on antibiotic stewardship. There is good evidence for using more oral antibiotics when possible, and to switch from intravenous/intramuscular to oral administration as soon as possible. Oral drugs are frequently just as effective and can lessen adverse effects of prolonged treatment and reduce the risk of resistance [10,11]. Moreover, several authors recommend switching to oral treatment as soon as possible in order to reduce the period of hospitalization and lower the risk of adverse effects as well as *Clostridioides difficile* infection [12,13]. Recently, Gamble et al. evaluated clinical outcomes of oral step-down antibiotics compared with continued intravenous therapy in UTIs [14]. In this study, which included 153 patients, the switch group was associated with reduced length of hospital stay and inpatient antibiotic costs [14]. The authors also highlighted the role of oral step-down treatment for antimicrobial stewardship. Recently, the results of the INSPIRE Randomized Clinical Trial have been published showing that computerized provider order entry prompts significantly reduced empiric extended-spectrum antibiotic use when compared with the standard antimicrobial stewardship recommendations [15].

### 3.3. Methodological Aspects

By definition, precision medicine means an integrated, personalized approach to each patient based on large-scale clinical data and the characteristics of the patients such as the clinical condition and history of treated infections or antibiotic use. We based our model on the concepts developed by Pizzuti et al. [16] and extended the model to cephalosporin-resistant and ESBL strains. Pizzuti argued that there is a greater chance of choosing the right antibiotic drug if a patient-specific risk factor evaluation is conducted [16]. The advantages are both clinical and economical. A risk factor-based approach leads to faster clinical improvement in patients and reduces the period of hospitalization and the likelihood of future antibiotic switches that may be required if the bacterial isolate in the urine is resistant to the prescribed antibiotic [16]. Moreover, this approach is able to reduce the risk of exposure to multiple classes of antibiotics for the treatment of the same infection, which may subsequently reduce the risk of colonization with multidrug-resistant pathogens. A precision model approach also reduces the risk of *Clostridioides difficile* infection, as well as the likelihood of developing adverse drug reactions [16]. Hopefully, AI will help us develop algorithms to assess all relevant variables as treatment decisions in UTI become more complex.

### 3.4. Strengths and Limitations of the Present Study

The use of a previously validated artificial intelligence-based model alongside the prospective protocol are regarded as strengths of this study. A limitation might be that the study was carried out in a single institution only. There is a risk of bias related to the qualifications of the urologists responsible for each study group. If the urologist who chaired the intervention group was better qualified, there is also a risk that the observed differences were not related to the precision model only but to the qualifications of the urologist in UTI treatment. There is also a risk of bias related to the observed hospitalization periods due to lack of oral equivalents for certain antibiotics, i.e., Amikacin, and the availability of urine culture results during weekends. We argue that our randomization process minimized selection bias, and Table 1 shows that there are no differences in comorbidities between the two groups. In the standard of care group, the most commonly prescribed antibiotics were piperacillin/tazobactam, followed by cephalosporins and fluoroquinolones. This may be because of a misinterpretation of the guidelines, which prefers wide-spectrum antibiotics without taking the patient’s risk factors into account, and because of a fear of ESBL strains. The choice of antibiotic was made when considering the local resistance patterns and while avoiding antibiotics with a high resistance rate (>20%) and other easy to collect clinical factors. A clearly stated null hypothesis and a total number of 80 and 77 patients in the two groups ensure robustness of our analyses. However, a larger study in several areas with different urologists is warranted to test the value of the precision medicine model used in the present study. Future studies should have a multicenter design, include creation of patient-specific antibiograms in intervention and control groups, and consider subgroup analysis by comorbidities and age.

## 4. Materials and Methods

### 4.1. Study Schedule and Patient Population

**Figure 1:**
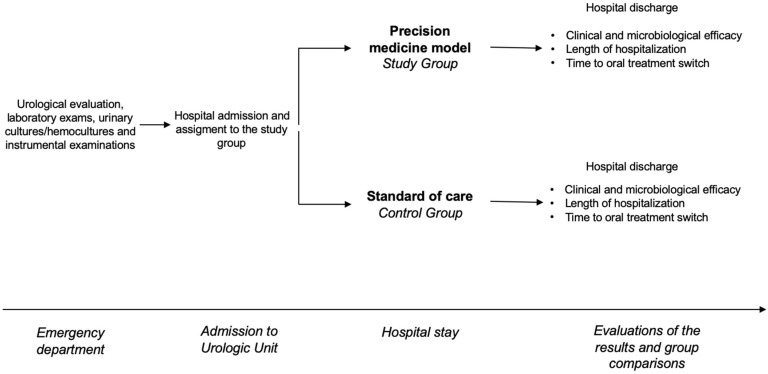
The figure shows the study schedule.

From January 2022 to March 2024, all patients admitted to the same urology unit with clinical suspicions of uAPN or cUTIs were enrolled in this trial. On arrival, all patients underwent a urological evaluation, laboratory exams, urinary cultures/hemocultures when indicated, and instrumental examinations. All patients were then treated with antibiotics. In the control group, empirical therapy was chosen according to the standard of care [3]. In the intervention group, the choice of empirical therapy at enrollment was made according to the precision medicine model using clinical tools to predict type of pathogen and likelihood of resistance [17]. The main outcome measures were the rates of clinical and microbiological success and the length of hospitalization. The time until switching to oral treatment was regarded as a secondary outcome measure. The study schedule is shown in Figure 1.

### 4.2. Clinical Presentations and Diagnostic Pathways at the Time of Enrollment

Patients were considered to have uAPN when presenting fever, chills, flank pain, nausea and/or vomiting, with or without the typical symptoms of cystitis [2]. Complicated UTI is defined according to EAU guidelines as an infection that occurs in an individual in whom factors related to the host or specific anatomical or functional abnormalities related to the urinary tract are believed to result in an infection that will be more difficult to eradicate than an uncomplicated infection [2]. All patients underwent urinalysis, urine culture, complete blood cell count, serum creatinine and blood urea nitrogen tests, and procalcitonin and C-Reactive Protein (CRP) level test while in the emergency department. Following the clinical urological examinations, all patients underwent complete abdominal ultrasound or computed abdominal tomography scan, in line with the urologist’s decision.

### 4.3. Choice of Empirical Antibiotic Treatment, Randomization, and Assignment to Study Groups

Shortly after hospital admission, all patients received empirical antibiotic treatment according to the study group assignment. Two equally qualified members of the urology team were responsible for the initial patient management. Patients were assigned to treatment groups according to a 1:1 randomization on the basis of the hospital arrival time. The randomization has been based on the assignment to each urologist. One urologist was asked to treat all patients with uAPN or cUTIs that he managed according to the standard of care. The other urologist was asked to treat all uAPN or cUTIs that he managed according to the precision medicine model by using clinical tools for the prediction of pathogens and antimicrobial resistance. Enrolled patients were not blinded. Hence, the urologist did not know what kind of patient he or she would encounter, and the patients did not know if he or she would be treated according to classical guideline recommendations or the new precision medicine principle. The standard of care treatment (control group) was defined as treatment suggested by EAU guidelines, fluoroquinolones, cephalosporines, aminoglycoside (with or without ampicillin), or an extended-spectrum cephalosporin or penicillin [2]. Whenever needed, an infectious disease specialist or clinical microbiologist was consulted. Selection of antibiotics in the intervention group was guided by a review published in 2023 by Pizzuti et al. which demonstrated that utilization of clinical tools for the prediction of antimicrobial resistance to fluoroquinolones, TMP-SMX, and third-generation cephalosporin improves ambulatory antibiotic management of uAPN [16]. Moreover, the precision model was modified by experience with previous predicting tools for antimicrobial resistance in uncomplicated UTIs [6,17]. We also considered local antibiotic resistance profiles of isolates while determining the most appropriate antibiotic to employ (Table 3). The choice to use amoxicillin/clavulanic acid was made due to the high sensitivity of the *Escherichia coli* and *Klebsiella pneumoniae* in our area, as well as the early switch to oral treatment. Analyses confirmed that the standard of care group followed the same local guidelines as the intervention group.

### 4.4. Development of the Precision Model Used in the Intervention Group

**Figure 2:**
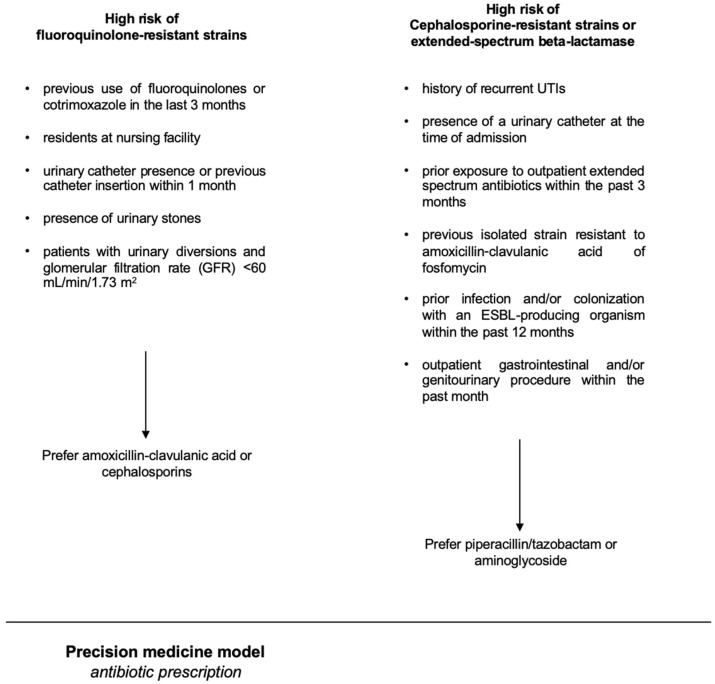
The figure shows the precision-medicine-based therapy of uAPN or cUTI.

A basic premise was the concept that the population-level antibiogram is different from the patient-specific antibiogram. There are lots of variables known to impact a patient’s risk of having a resistant organism, such as prior antibiotic exposure, prior findings on urine culture, a history of resistant pathogens, a history of UTI, the patient`s age, and the presence of a bladder catheter or a nephrostomy [18]. As independent risk factors predicting fluoroquinolone-resistant strains, we considered the following parameters: previous use of fluoroquinolones or cotrimoxazole in the last 3 months, being a resident at a nursing facility, presence of a urinary catheter or a history of a catheter insertion within the previous month, presence of urinary stones, and patients with urinary diversions and renal insufficiency (defined as glomerular filtration rate (GFR) < 60 mL/min/1.73 m^2^). As independent risk factors for having a uropathogenic strain resistant to cephalosporines or an extended-spectrum beta-lactamase (ESBL) strain, we considered the following parameters: a history of recurrent UTIs, the presence of a urinary catheter at the time of admission, and prior exposure to outpatient extended-spectrum antibiotics within the past 3 months, previous isolation of strains resistant to amoxicillin-clavulanic acid or fosfomycin, prior infection and/or colonization with an ESBL-producing organism within the past 6 months [19], and an outpatient gastrointestinal and/or genitourinary procedure within the previous month. The precision-medicine-based therapy of uAPN or cUTI in hospitalized patients is outlined in Figure 2.

### 4.5. Outcome Measures

A clinical, urological evaluation was carried out at hospital admission and immediately before hospital discharge. Microbiological analysis was carried out at hospital admission and discharge and was repeated in case of treatment failure. Clinical success was defined as complete resolution of signs and symptoms of infection on cessation of the antibiotic treatment [2]. Microbiological success was defined as the absence of bacterial growth or <10^2^ colony-forming units (CFU) of the original pathogen in post-treatment urine culture [2]. Treatment failure was defined as the persistence of symptoms or increase of symptom severity. The length of hospitalization was defined as the number of days a patient remained in hospital during a single admission [20]. The time to switch to oral treatment was defined as the number of days from the start of intravenous antibiotic treatment until the start of oral administration of antibiotics and cessation of the intravenous treatment. The switch to oral treatment was based on the clinical response and on the reduction of inflammation markers. We chose “time to oral switch” as a secondary outcome instead of a primary outcome because: (i) the clinical and microbiological success and the length of hospitalization have more significant impact on patients’ overall outcome, and (ii) the time to oral switch is not always correlated with the length of hospitalization.

### 4.6. Microbiological and Laboratory Examinations

The microbiological and chemical laboratory analyses used for patients with suspected UTIs in our department have been described by us in previous studies [21]. A colony count of ≥10^5^ CFU/mL was considered the cut-off value for a significant microbiological finding of the same isolated strain [2].

### 4.7. Statistical Analysis and Ethical Considerations

As null hypothesis, we considered that there was no difference between the two groups in terms of rate of clinical and microbiological success and length of hospitalization. In order to obtain statistical significance, sample size estimation was based on the following assumptions: difference in terms of the clinical and microbiological successes between the two groups: 30%; α error level, 0.05 two-sided; statistical power, 80%; anticipated effect size, Cohen’s d = 0.5; difference in terms of the length of hospitalization between the two groups: −2 ± 1; α error level, 0.05 two-sided; statistical power, 80%; anticipated effect size, Cohen’s d = 0.5. The calculation yielded 70 evaluable individuals in each group. Considering a drop-out rate of 10%, the final sample size was set at 77 patients. Statistical analysis was performed as follows: continuous variables were presented as mean and standard deviation and categorical variables were presented as absolute (*n*) and relative (%) frequency distributions. *T* tests were used to compare average performances between hospital admission and hospital discharge. The threshold of statistical significance was set at *p* < 0.05. All reported *p*-values are two-sided. All statistical analyses were performed using SPSS 23.0 (IBM Corporation, Armonk, NY, USA). Ethical review and approval were waived for this study, as all patients underwent usual management of urinary tract infections with antimicrobial therapy. No new drug has been tested. However, all anamnestic, clinical, and laboratory data containing sensitive information about patients were de-identified to ensure analysis of anonymous data only and blinding of the trialists performing the analysis. The de-identification process was performed by non-medical staff by means of dedicated software (PhysioNet; 2019) [22]. According to Italian law, this study did not require written informed consent from patients. The study was, however, conducted in line with the Good Clinical Practice guidelines and the ethical principles laid down in the latest version of the Declaration of Helsinki.

## 5. Conclusions

The findings in our study must be interpreted in the context of the rapidly increasing problems related to antibiotic resistance in urological settings and the need for increasing the adherence of urologists and other physicians managing UTIs to the principles of antimicrobial stewardship [23]. We showed that for hospitalized patients with uAPN and cUTIs, use of a precision medicine model resulted in shorter hospital stays, quicker transition to oral therapy, and less need for antibiotic treatment re-evaluation and modification, ultimately contributing to a more rational use of antibiotics and to contrast AMR. We argue that our precision medicine model pays more attention to local resistance rates and might enhance our adherence to antimicrobial stewardship principles in the management of patients with uAPN and cUTIs in daily clinical practice.

## Figures and Tables

**Table 1:** Anamnestic, clinical, and laboratory characteristics of all patients at the time of enrollment. The table shows all demographic, anamnestic, clinical, and laboratory data at enrollment; *n*° = number; SD* = standard deviation.

	Study Group	Control Group	*p*
	Mean (SD* or %)	Mean (SD* or %)	
Patients (*n*°)	77	80	
Age	65.8 ± 8.1	67.1 ± 9.0	0.34
Sex			
Male	47 (61.1)	49 (61.3)	0.99
Female	30 (38.9)	31 (38.7)	
Comorbidities			0.52
Hypertension or Cardiovascular diseases	43 (55.8)	49 (61.2)	
Diabetes mellitus	12 (15.6)	15 (18.7)	
No	22 (28.6)	16 (20.1)	
Clinical presentation			0.85
uAPN	17 (22.1)	19 (23.7)	
cUTI	60 (77.9)	61 (76.3)	
fever and symptoms	77 (100)	80 (100)	-
symptoms, only	-	-	
Inflammation markers			
C-reactive Protein (mg/L)	156.8 ± 38.3	163.7 ± 40.7	0.27
Procalcitonin	3.5 ± 2.4	4.3 ± 3.9	0.12
White blood cells (×10^3^/mm^3^)	17.291 ± 2.127	16.573 ± 3.740	0.14
Isolated strain (10^5^ CFU/mL)			0.99
*Escherichia coli*	65 (84.5)	68 (85.0)	
*Enterococcus* spp.	2 (2.6)	1 (1.3)	
*Klebsiella* spp.	7 (9.1)	9 (11.2)	
Other	3 (3.8)	2 (2.5)	
Empirical antibiotics prescribed			
Amikacin	5 (6.5)	-	
Amoxicillin-clavulanic acid	42 (54.8)	3 (3.7)	
Ampicillin	1 (1.3)	-	
Ceftriaxone	8 (10.1)	13 (16.4)	
Ceftazidime	4 (5.2)	1 (1.3)	
Cefepime	-	-	
Ciprofloxacin	2 (2.6)	3 (3.7)	
Gentamicin	7 (9.1)	-	
Levofloxacin	1 (1.3)	3 (3.7)	
Meropenem	2 (2.6)	2 (2.5)	
Piperacillin/tazobactam	5 (6.5)	55 (68.7)	

**Table 2:** Clinical and microbiological results at the follow-up evaluations according to the study group. The table shows all clinical and microbiological results at the follow-up evaluations according to the study group. SD* = standard deviation.

	Study Group	Control Group	*p*
	Mean (SD* or %)	Mean (SD* or %)	
Clinical success	72 (93.5)	75 (93.7)	0.97
Time to defervescence (h)	81 ± 11	78 ± 12	0.12
Time to inflammation markers halving (h)	19 ± 12	21 ± 11	0.27
Microbiological success	74 (96.1)	76 (95.0)	0.99
Antibiotic change	6 (7.8)	12 (15.0)	0.04
lack of clinical response	4 (66.6)	9 (75.0)	
lack of inflammation markers halving	2 (33.4)	3 (25.0)	
Time to oral treatment switching (h)	72 ± 4	96 ± 8	<0.001
Amikacin	70 ± 2	-	
Amoxicillin-clavulanic acid	65 ± 1	64 ± 3	
Ampicillin	68 ± 2	-	
Ceftriaxone	75 ± 3	81 ± 3	
Ceftazidime	70 ± 1	70 ± 9	
Cefepime	-	-	
Ciprofloxacin	69 ± 2	71 ± 3	
Gentamicin	69 ± 3	-	
Levofloxacin	70 ± 1	70 ± 7	
Meropenem	77 ± 4	76 ± 3	
Piperacillin/tazobactam	78 ± 3	99 ± 5	
Length of hospitalization (days)	5 ± 2	8 ± 3	<0.001

**Table 3:** Local antibiotic resistance profiles of most common isolates from urine cultures. The table shows the local antibiotic resistance profile of most common isolates from urine cultures.

	Sensibility (%)	
	*Escherichia* *coli*	*Klebsiella* *pneumoniae*
Antibiotics		
Amikacin	98.5	98.7
Amoxicillin-clavulanic acid	86.8	87.5
Ceftriaxone	82.8	80.1
Ceftazidime	86.1	81.8
Cefepime	86.3	82.9
Ciprofloxacin	78.7	87.6
Cotrimoxazole	77.9	83.5
Colistin	99.2	99.0
Gentamicin	90.5	95.3
Meropenem	99.9	98.3
Piperacillin/tazobactam	96.0	89.9

## Data Availability

Data are not available due to the restriction of Italian bylaw on privacy.

## Abbreviations

The following abbreviations are used in this manuscript:

UTIs	Urinary Tract Infections
uAPN	acute uncomplicated pyelonephritis
cUTIs	Complicated Urinary Tract Infections
EAU	European Association of Urology
AMR	Antimicrobial Resistance
WHO	World Health Organization
ESBL	Extended-Spectrum Beta-Lactamase
AI	Artificial Intelligence
CRP	C-Reactive Protein
